# Enhancing Substance Use Disorder Recovery through Integrated Physical Activity and Behavioral Interventions: A Comprehensive Approach to Treatment and Prevention

**DOI:** 10.3390/brainsci14060534

**Published:** 2024-05-24

**Authors:** Yannis Theodorakis, Mary Hassandra, Fotis Panagiotounis

**Affiliations:** 1 Department of Physical Education and Sport Science, University of Thessaly, 42100 Trikala, Greece; mxasad@uth.gr; 2 Therapy Center for Depended Individuals, 11636 Athens, Greece; panagiotounisfotis@gmail.com

**Keywords:** substance use disorder, addiction therapy, exercise integration, holistic approach, physical activity, sports

## Abstract

The global issue of substance abuse demands ongoing initiatives aligned with the United Nations Sustainable Development Goals. With drug use remaining prevalent worldwide, interventions are critical to addressing the associated health challenges and societal implications. Exercise and physical activities have emerged as integral components of substance use disorder (SUD) treatment, offering promising avenues for prevention, intervention, and recovery. Recent research underscores the efficacy of exercise in reducing substance cravings, promoting abstinence, and improving overall well-being. However, integrating exercise into SUD recovery programs presents challenges such as dropout rates and cultural considerations. This paper synthesizes existing literature on exercise integration into SUD recovery, highlighting strategies for enhancing treatment outcomes and addressing barriers to exercise adherence. Drawing on cognitive–behavioral therapy, experiential learning, motivational interviewing, and goal-setting techniques, the holistic approach outlined in this paper aims to empower individuals both mentally and physically, fostering resilience and supporting long-term recovery. In conclusion, new initiatives need to be taken by advocating for inclusive policies, promoting community engagement, and fostering collaborations across sectors. By doing so, stakeholders can optimize the effectiveness of exercise programs and contribute to sustainable rehabilitation efforts for individuals with SUD.

## 1. Introduction: Global Substance Abuse Trends

In the vast issue of substance abuse on a global scale, ongoing initiatives and strategic directions are continuously taken. In alignment with the United Nations Sustainable Development Goals, governments have committed to enhancing the prevention and treatment of substance abuse [1]. Worldwide, drug use remains prevalent and the estimated number of drug users grew from 240 million in 2011 to 296 million in 2021 (5.8% of the global population aged 15–64) [2]. The World Health Organization underscores the severity of the issue, and the emergence of synthetic opioids has contributed to an increase in mortality trends, particularly in high-income countries [3].

Drug addiction is a complex chronic disease impacting the brain’s reward, motivation, and memory circuits, leading to biological, psychological, social, and spiritual manifestations. Substance use disorders (SUD) cause significant distress, escalating use, craving tolerance, and social harm associated with unhealthy lifestyle choices and high rates of relapse. The United Nations Office for Drugs and Crime has stated that sport could be a powerful tool to engage communities and prevent drug use [4,5].

This study aims to focus not only on the use of sports and physical activities for patients with SUD but also on the integration of these activities into the therapeutic process and treatment. This integration requires the utilization of effective psychological strategies within the therapeutic process to enrich the traditional practice of treatment in addiction recovery. All of the above have been presented fragmentarily in the relevant literature and have not been systematically recorded and presented until now. Policy recommendations have not been formulated in the literature. Based on standardized methodology and selection criteria, 23 systematic reviews and meta-analyses from recent years were recorded and analyzed.

## 2. The Role of Exercise in SUD Treatment

### 2.1. Overview of Current Research

Exercise and physical activities play a pivotal role in addressing the issue of SUD, as evidenced by recent advancements in research. Numerous studies have documented the efficacy of exercise and physical activities in reducing cravings for substance use, promoting abstinence, improving quality of life, and acting as valuable components for prevention and intervention, complementing traditional therapy [6,7,8,9,10].

### 2.2. Benefits of Physical Activity in SUD

Several reviews and meta-analyses have documented the importance of physical activity and exercise programs in the treatment of addictions in various age groups and settings [10,11,12,13,14,15,16,17,18]. In general, intervention programs have shown positive effects, encompassing a reduction in anxiety and depressive symptoms, enhanced working memory, increased self-confidence, improved self-esteem, positive changes in body image, mood enhancement, elevated well-being and quality of life, alterations in behavior, and positive lifestyle changes. Systematic reviews have indicated improved substance use outcomes in young people, including a reduction in the frequency of use, amount of use, intent to use, and/or cravings, as well as improved physical fitness and quality of life in the treatment of addiction to alcohol, tobacco, and other substances [19,20].

It seems that the positive impact of participation in exercise programs and physical activities on adopting a healthy lifestyle and fostering behavior change has been documented. With recent research and meta-analyses emphasizing the positive outcomes of exercise for individuals with SUD, it is now imperative to systematically record, organize, and integrate the knowledge acquired to date into corresponding therapeutic programs in a creative manner. However, the majority of the published literature consists of studies on small samples, presenting an opportunity for future investigators to validate findings with larger sample sizes and explore different exercise modalities [18]. The new challenge is how to integrate therapeutic treatment and exercise programs into a unified therapeutic process while also incorporating counseling therapy and structured exercise programs.

## 3. Integrating Exercise with Therapeutic Approaches

The integration of sports into a therapeutic program represents a holistic approach aimed at fostering responsibility, self-awareness, strengthened self-efficacy, coping skills, relationship building, and cooperation. Additionally, the sporting environment may enforce the application of learned skills to real-life situations, facilitate personal development, and support re-entry into society [21].

### 3.1. Types of Activities

A review of studies has shown that preferred types of activities, including aerobic exercises, strength training, running, walking, mind–body exercises, and outdoor sports adventure activities are all acceptable options for SUD recovery. Walking is suggested as one of the most popular activities and a safe choice for clients in poor physical condition or with limited motor function [10,22,23,24]. Maximal strength training is a feasible, safe, and effective method to improve muscle strength and function [25]. In addition, yoga programs significantly reduce anger, confusion, depression, fatigue, and tension, while significantly increasing relaxation, acting as an effective complementary treatment modality in the management of patients with SUD [26,27]. Tai Chi and Qigong exercise and traditional Chinese health-promoting exercise have had positive effects on physical and mental health, quality of life, sleep quality, depression, anxiety, and withdrawal symptoms [28,29,30,31].

### 3.2. Exercise Intensity

Individuals undergoing SUD recovery, facing significant physical and psychological challenges, especially in the early stages of recovery due to their poor quality of life and sedentary lifestyles, related to drug abuse, need to carefully address the intensity of their exercise routines [32,33]. High-intensity programs have low compliance rates compared to low- and moderate-intensity programs. In the early stages of treatment, voluntary participation in walking and low-to-moderate-intensity exercise is recommended, as these have protective effects and reduce the risk of injury or other side effects. Moderate-intensity aerobic exercise interventions, including cycling, jogging, callisthenics, and stretching, are effective treatments for methamphetamine-dependent individuals, improving their social, physical, and mental health [34]. Additionally, moderate-intensity exercise has a higher rate of adherence than intense exercise [35,36,37]. A systematic review revealed that the prevailing physical activity intervention involved moderate intensity and was conducted three times per week. Additionally, there was a reported decrease in depressive symptoms [17]. In contrast, the evidence supporting the effects of anaerobic exercise on SUD is weak [12].

### 3.3. Sports Involvement

While the role of sports is generally associated with positive health outcomes, individuals engaged in competitive sports may face challenges related to the culture, expectations, and demands of the sport, creating a context in which SUD might develop. The mechanisms leading to substance use and addiction activation include personal characteristics such as low self-esteem, high competitiveness, and a lack of effective coping strategies [38]. However, the implementation of team and individual sports in addiction treatment does not encounter such challenges, and their roles differ. For example, in the initial stages, modifications to the rules of the game are recommended, gradually evolving into their standard forms. For instance, activities involving a soccer ball can be initially designed to instill the principles of cooperation, collaboration, and team building, before progressing into a full-fledged soccer game adhering to standard rules.

### 3.4. Life Skills

Physical activities and exercise enhance overall physical health, fitness, and well-being, contributing to better mental health for individuals in recovery. Integrating life skills through exercise and physical activities into a recovery program helps in transfer to the challenges of everyday life, and reinforcing the therapeutic process. These life skills include goal-setting, time management, emotional regulation, interaction, leadership problem-solving, role modeling, and decision-making. It is crucial to maintain continuity and sequence over time, establishing a routine so that participants can effectively transfer these acquired skills into their daily lives. Sports programs offer assistance in fostering and transferring life skills for socially vulnerable adults [39].

### 3.5. Cooperative and Problem-Solving Activities

Games, trust-building, and cooperative physical activities contribute to positive therapeutic outcomes by promoting participant interaction, communication, teamwork, group cohesion and cooperation among participants, fostering a sense of community, and providing opportunities for participants to experience emotional support, responsibility and safety. Initiative, problem-solving, and decision-making activities aligned with therapeutic goals contribute to a holistic therapeutic experience. These activities often involve coping strategies, responsibility-taking, and self-awareness. Practicing problem-solving abilities gradually can lead to the successful resolution of real-life problems that may be applied in daily life, thus assisting the SUD recovery [40].

### 3.6. Adventure Therapy

High-adventure activities in natural environments expose individuals to controlled challenges. Accomplishing outdoor activities, such as rock climbing, hiking, overnight camping, backpacking, rafting, rock climbing, caving, and other outdoor pursuits enhances self-esteem and confidence. Adventure therapy employs the natural environment and adventure-based sports activities as an experiential, interactive model, aiding in emotional healing. Research indicates that adventure therapy is a viable and promising treatment approach by fostering autonomy, life skills, a sense of accomplishment and a sense of responsibility in real-life situations [23,41,42].

The integration of exercise and physical activities within therapeutic approaches for SUD recovery is a multifaceted strategy that offers comprehensive benefits across physical, psychological, and social dimensions. By incorporating a diverse range of activities, including aerobic exercises, strength training, mind–body practices, and adventure therapy, this approach supports the development of crucial life skills, enhances mental health, and fosters a sense of community and personal accomplishment. Moreover, the careful calibration of exercise intensity ensures that individuals at various stages of recovery can participate safely and effectively, maximizing adherence and minimizing risks. As such, these integrated exercise programs do not merely serve as adjunct treatments but are pivotal in holistic rehabilitation efforts, promoting sustained recovery and a higher quality of life. Through strategic implementation and supportive environments, these programs empower individuals with SUD to reclaim control over their lives, paving the way for successful reintegration into society and ongoing personal development.

## 4. Integrating Exercise into SUD Recovery: Cognitive–Behavioral Strategies for Holistic Rehabilitation

Addiction is a multifaceted disorder influenced by a complex interplay of external circumstances, psychological states, and personal traits. These factors converge to foster both conscious and unconscious drives that compel individuals to seek pleasure or avoid discomfort through substance use. The consequences of long-term drug use are profound, often leading to unemployment, strained family relationships, and a deterioration of social networks. In addressing these challenges, integrating exercise programs and physical activities into SUD recovery strategies offers a promising avenue to disrupt the cycle of addiction. This integration aims to replace the pursuit of drug-related pleasure with healthier physical activities that activate rewarding neural pathways, thereby establishing new patterns of behavior that support both mental and physical well-being. By incorporating cognitive–behavioral therapy (CBT) and tailored exercise regimes, this approach not only targets the symptoms of SUD but also empowers individuals to regain control over their lives, promoting sustainable recovery and an enhanced quality of life.

### 4.1. Cognitive–Behavioral Therapy (CBT)

CBT for addiction is an umbrella term encompassing interventions that employ a range of cognitive and behavioral techniques, demonstrating efficacy as part of combination treatment strategies. The utilization of healthier coping strategies is crucial for recovery from substance use disorders. This includes incorporating elements such as education, skills training, and behavioral strategies to avoid triggers, set goals, utilize social support, learn effective communication skills, behavioral regulation, and engage in social comparison. Various forms of exercise serve as examples of healthy coping strategies frequently integrated into CBT treatment [43,44,45,46].

### 4.2. Motivation

Despite the positive effects of regular physical activity on treatment outcomes and risk factors for relapse, lack of motivation is one of many perceived barriers to initiating exercise that contribute to poor adherence to interventions. The high rates of coexisting mental disorders which characterize this population negatively affect motivation and cause patients to drop out of exercise [35]. Exercise interventions that combine motivational interviewing, a client-centered approach designed to enhance intrinsic motivation and resolve ambivalence towards change, and contingency management are associated with the initiation and maintenance of exercise behaviors [33]. Recovery focuses on strengthening individuals’ skills and abilities and supporting the enhancement of their motivation to increase the likelihood that they will commit to a specific behavioral change plan. Thus, the provision of choice of sports and exercise activities, the setting of new challenging goals, continuous feedback, support from peers, and the self-monitoring of performance may lead to the enhancement of positive behaviour change [32,40,47]. Autonomy support and respect for individual preferences in exercise types, the reinforcement of physical and mental abilities, social inclusion, and social support play a crucial role in achieving positive outcomes in SUD recovery [14,48,49,50].

### 4.3. Self-Efficacy

Engaging in exercise can empower individuals facing drug addiction, enabling them to attain improved physical fitness and enhance their psychological well-being, encompassing aspects like self-efficacy and willpower. Self-efficacy refers to individuals’ ability to initially decide to make behavioral changes and successfully adhere to specific healthy behaviors, such as exercise programs [51]. Exercise self-efficacy and quality of life were found to be negatively correlated with relapse tendency. Establishing positive exercise habits and adherence proves advantageous not only in preventing relapse but also in elevating the overall quality of life of individuals grappling with drug addiction [52]. According to the theory, self-efficacy is reinforced through mastery experiences, vicarious experiences, verbal persuasion, and emotional and physiological states. Psychological techniques for enhancing self-efficacy include regular practice, realistic goal-setting, continuous evaluation, trying new things, finding a role model, providing positive feedback and support, promoting autonomy, creating a positive atmosphere, and maintaining a positive attitude. A moderate-intensity exercise program, employed as an adjuvant treatment alongside a cognitive–behavioral model for alcohol use disorder intervention, proved effective in reducing depression and anxiety while increasing self-efficacy [45].

### 4.4. Attitudes

Exercise can change individuals’ attitudes towards drug use, enhance their willpower and desire to detoxify or develop drug resistance, and prevent relapse. Positive attitudes and intentions towards substance abuse treatment are related to abuse treatment completion. A relevant study tested the model of planned behavior and predicted substance abuse treatment completion. Attitude and control components independently predicted intention which positively associated with treatment completion [53]. Professionals and treatment providers should encourage beliefs, attitudes, moral norms, and perceived behavior control to increase intentions among future participants in addiction treatment [54,55]. Thus, the implementation of attitude change strategies is helpful in SUD recovery.

### 4.5. Goal Setting for Combining Therapeutic and Exercise Goals

Goal setting can be used as an effective motivational treatment in addiction therapy to help clients improve work, education, health, and nutrition, and also to learn new skills or improve old ones in areas such as sports, art, music, and hobbies. Client motivation is a dynamic process of responding to a variety of interpersonal influences including advice, feedback, goal setting, contingencies, and perceived choice among alternatives [56]. Goal setting and goal monitoring are collaborative processes where clinicians and clients work together to identify and formulate therapeutic goals. Relevant research and reviews have highlighted crucial principles and practices, emphasizing that goal setting and monitoring serve as a collaborative method for mapping and tracking a course of treatment [20,40,57,58]. As in the field of sports and exercise, goal-setting theory is particularly popular and effective. The combination of therapeutic and athletic goals can likely help individuals struggling with addiction to lead a more active and healthier lifestyle integrated into their daily routines.

### 4.6. Experiential Learning

A promising environment for recovery programs is facilitated through an approach known as experiential learning. Experiential learning is an educational method that underscores real-world experiences to acquire knowledge, develop skills, and foster personal growth. To achieve this, outdoor leisure activities or adventure therapy activities, should be adapted through experiments and real-life problem-solving tasks. Learners deepen their understanding of the subject matter and are encouraged to reflect on what happened, what they learned, and how it connects to their therapeutic goals. Learners work together, share ideas, and learn from one another, which helps them understand their strengths and areas for improvement. Instead of receiving direct orders, learners are encouraged to discover and participate in group projects, and simulations of real-world situations co-constructing their experiences, enhancing self-awareness about their decisions and outcomes, and integrating routines and monitoring into their practice during daily tasks in wilderness settings. Research indicated that therapeutic outdoor interventions have increased in popularity as alternative approaches for treating at-risk or adjudicated adolescents [41,59,60].

### 4.7. Adaptations to Participants

A systematic review revealed that when individuals embrace exercise and sports participation as new habits, they experience positive emotional effects, sidestep negative emotions, and play a role in self-defense and self-protection [16]. A relevant review showed that physical activity has been incorporated into treatments in combination with other pharmacological or behavioral treatments. Healthy nutrition to achieve optimal health and well-being should also enrich these new habits [11]. Adapting to the participants’ levels is crucial. Adjusting activities to the treatment program’s duration, and aligning with clients’ developmental positions, therapeutic goals, and dynamics is necessary. Additionally, adopting measures such as reinforcing, monitoring physical and emotional safety, and conducting regular and safe activities in a controlled environment is recommended [32,33].

### 4.8. Adherence

Engaging individuals with SUD in exercise programs is a challenging procedure, with dropout rates exceeding 50%. Poor social support for exercise, financial difficulties, low self-efficacy, lack of time, and low motivation to exercise, combined with poor initial physical condition, act as barriers to retention in exercise programs. Social support, increased self-efficacy, appropriate physical activity choices, goal setting, positive reinforcement, and feedback are all important factors in enhancing adherence to an exercise program [61]. Higher body mass index and lower educational attainment are important factors that decrease adherence to physical exercise participation in individuals with SUD [62]. Adherence to exercise programs can be achieved through appropriate safety protocols. For therapists, it is essential to respect participants’ cultural beliefs and values related to collaboration, emotional management, conflict resolution, personal expression, and physical contact. Integrating cultural considerations is pivotal for creating an inclusive and respectful environment.

## 5. Conclusions: Enhancing SUD Recovery through Holistic Exercise Integration

The integration of exercise and physical activities into substance use disorder (SUD) recovery programs represents a transformative approach that extends beyond traditional treatment modalities. By combining cognitive–behavioral therapy (CBT) with structured physical activities, the approach addresses both the psychological and physical facets of addiction. This dual focus not only disrupts the detrimental cycle of substance dependency but also cultivates a new paradigm of wellness and self-efficacy.

Key elements such as motivation enhancement, goal setting, and experiential learning are crucial. They bolster the treatment framework by promoting consistent engagement and fostering a sense of achievement. Moreover, adapting exercise regimes to fit individual recovery phases and capabilities ensures that each participant can engage safely and effectively, maximizing therapeutic outcomes.

By fostering positive attitudes, enhancing motivation, and supporting the development of healthy habits, this holistic strategy significantly contributes to the likelihood of recovery success. Furthermore, by integrating coping strategies within the framework of regular physical activity, individuals are equipped with the tools necessary to manage triggers and stressors effectively, thereby reducing the risk of relapse.

In essence, the implementation of exercise and physical activity within SUD treatment protocols not only aids in the physical rejuvenation of individuals but also serves as a pivotal component in psychological rehabilitation. This integrative approach underscores the necessity of treating both the body and the mind to facilitate a comprehensive recovery process, ultimately leading to improved quality of life and long-term sobriety. Thus, it is imperative for treatment programs to incorporate these strategies as standard practice, reflecting an evolving understanding of addiction recovery as a multidimensional process.

## 6. Recommended Strategies for Enhancing Treatment Outcomes and Social Inclusion

Despite advances in our understanding and management of SUDs, there remains a need to develop policies ensuring support and access to prevention and treatment [63]. The following recommendations are grounded in a thorough synthesis and analysis of the current literature on integrating exercise into SUD treatment. These recommendations are informed by the gaps, trends, and insights identified through the review process, aiming to contribute valuable insights to the field by offering practical implications for research directions and clinical practice.

Global and European policies. The European Union [64], in line with the United Nations 2030 Agenda for Sustainable Development, has recognized that stakeholders and sports organizations can use innovative social, organisational, political, digital, and technological approaches are necessary to tackle existing and emerging threats and challenges relating to various vulnerable groups and among them abuses.

Role of Health Organizations. Health organizations, ministries of health, and other federations play a crucial role in implementing exercise programs for the inclusion of addicts in local communities, and they must be more activated towards this direction. Leveraging their knowledge, influence, and experience, these organizations can spearhead initiatives that promote sports and physical activities as integral components of addiction recovery. The societal acceptance of the role of sports in providing hope for inclusion and improving mental health and well-being will enforce SUD treatment programs.

Cross-sector collaboration. Collaboration among experts from various fields is crucial, promoting parallel practices and cooperation among therapists, health professionals, and exercise specialists. This collaboration ultimately enhances confidence and proficiency in implementing exercise programs for SUD. In an innovative intervention, therapeutic organizations are recommended to involve their entire staff and foster a secure environment, offering equal opportunities to all individuals and contributing to their treatment and social reintegration.

Comprehensive involvement. Therapeutic organizations should prioritize the integration of sports programs into standard treatment protocols, by identifying behavior change techniques that will enhance SUD treatment strategies. Also, it is suggested to increase the time spent on sports and exercise programs. Combining traditional addiction therapy approaches with the contributions of exercise programs can help overcome obstacles, reinforce positive attitudes toward treatment, motivate adherence, and increase social interaction with friends, family, and the community, ultimately creating a more supportive living environment.

Community programs. Policymakers, health organizations, and governments at the national and international levels should establish community engagement programs that promote awareness of the benefits of sports and exercise in addiction recovery.

Reducing stigma. These programs can include public events, workshops, and educational campaigns aimed at changing societal perceptions and reducing stigma. Integration into educational curricula, public awareness campaigns, collaboration with sports organizations, and fostering partnerships between health organizations and sports clubs, leagues, and associations are essential.

Policy advocacy for inclusive sports programs. Advocacy for policies that promote the inclusion of individuals in addiction recovery in mainstream sports programs at the local, regional, and national levels is crucial. Encouraging collaboration between the healthcare sector, sports industry, education, and local businesses is necessary to create a comprehensive, cross-sector approach.

Further research in the area. Excessive internet use in adolescents has been associated with emotion dysregulation, internet addiction, increased BMI levels, and symptoms of social anxiety [65,66]. Physical activity would be helpful for internet addiction for university students, by increasing coping styles [67]. A meta-analysis indicated that adolescents with low levels of self-esteem, self-control, and social support were more likely to develop smartphone addiction [68]. A review of intervention studies on digital addiction in children and adolescents revealed that most interventions were cognitive–behavioral therapies. Although these studies have small sample sizes and short intervention durations, resulting in limited generalizability, involvement in physical activities was suggested [13,69].

In general, this area of research needs further well-designed trials. An optimal creative practice would be for professional organizations to collaborate with academic institutions and establish standardized training programs for therapists and healthcare professionals. A meta-analysis identified a wide range of methodological concerns for the clinical recommendation of exercise treatment [70]. Assessing motivational strategies for and perceptions of exercise involvement and identifying perceived barriers and facilitators of exercise participation is a vital step in devising effective exercise programs to stimulate more effective recovery outcomes as well as physical and mental health benefits [15]. Moreover, as substance abuse and addiction likely occur at a lower rate in athletes compared to the general population, there is a need for more rigorous, high-quality studies on how to approach this vulnerable subset of the population [71].

SUD is characterized by cognitive impairment, and executive function is a key factor in determining treatment outcomes, including dropout rates and the risk of relapse post-treatment. It seems that aerobic exercise programs have beneficial effects on cognitive functions, emotions, cravings, and physical fitness among SUD patients [72,73,74]. While physical activity is believed to have a positive impact on cognitive function, there is still a gap in understanding the potential benefits of aerobic exercise specifically for executive function in SUD treatment. This area requires further investigation in upcoming research [75].

It seems that exercise stimulates the release of endorphins and neurotransmitters that improve mood, leading to feelings of euphoria and well-being. Incorporating exercise into SUD treatment can naturally enhance mood, help manage emotional distress, and reduce relapse risk. Structured physical activities can improve cognitive performance, aiding recovery and sobriety. Exercise also mitigates neurodegenerative effects from chronic substance use, supporting brain recovery by creating healthier neural pathways and promoting cognitive flexibility [48,76,77].

## 7. Conclusions

The implementation of exercise programs, designed to impart life skills through cooperative trust and support activities, as well as high adventure activities, is correct to be applied and tailored to the participants’ individual levels and personal preferences. The proposed intervention programs prioritize comprehensive skill development, encompassing goal-setting, time management, emotional control, communication, social interaction, leadership, problem-solving, and decision-making—all seamlessly integrated into the context of the treatment protocols. The experiences gained through these programs will empower participants to confront the realities they have lost due to substance abuse. The role of governmental bodies and recovery agencies in the implementation of the suggested policy recommendations is pivotal in assisting at-risk populations. Through exercise programs and active participation in physical activities, these entities contribute to the revitalization of individuals, fostering social inclusion, the adoption of a healthy lifestyle, and achieving recovery.

## Data Availability

The data presented in this study are available on request from the corresponding author. The data are not publicly available, because of privacy.
